# On Parallel Streams through the Mouse Dorsal Lateral Geniculate Nucleus

**DOI:** 10.3389/fncir.2016.00020

**Published:** 2016-03-30

**Authors:** Daniel J. Denman, Diego Contreras

**Affiliations:** ^1^Allen Institute for Brain ScienceSeattle, WA, USA; ^2^Perelman School of Medicine, University of PennsylvaniaPhiladelphia, PA, USA

**Keywords:** LGN, parallel processing, mouse vision, cell types, mouse models

## Abstract

The mouse visual system is an emerging model for the study of cortical and thalamic circuit function. To maximize the usefulness of this model system, it is important to analyze the similarities and differences between the organization of all levels of the murid visual system with other, better studied systems (e.g., non-human primates and the domestic cat). While the understanding of mouse retina and cortex has expanded rapidly, less is known about mouse dorsal lateral geniculate nucleus (dLGN). Here, we study whether parallel processing streams exist in mouse dLGN. We use a battery of stimuli that have been previously shown to successfully distinguish parallel streams in other species: electrical stimulation of the optic chiasm, contrast-reversing stationary gratings at varying spatial phase, drifting sinusoidal gratings, dense noise for receptive field reconstruction, and frozen contrast-modulating noise. As in the optic nerves of domestic cats and non-human primates, we find evidence for multiple conduction velocity groups after optic chiasm stimulation. As in so-called “visual mammals”, we find a subpopulation of mouse dLGN cells showing non-linear spatial summation. However, differences in stimulus selectivity and sensitivity do not provide sufficient basis for identification of clearly distinct classes of relay cells. Nevertheless, consistent with presumptively homologous status of dLGNs of all mammals, there are substantial similarities between response properties of mouse dLGN neurons and those of cats and primates.

## Introduction

In carnivores and primates, processing of visual information is carried out in parallel streams from the retina to the cerebral cortex (Stone, 1983; Livingstone and Hubel, 1988; Merigan and Maunsell, 1993; Wässle, 2004; Nassi and Callaway, 2009) Different types of visual information remain segregated in these pathways, and are later combined in cortex for different visual processing tasks.

The first evidence that visual pathways are organized into parallel streams was the recording of early and late components in the compound action potential triggered by electrical stimulation of the optic nerve of frog and rabbit (Bishop, 1933). The discovery of the presence in the optic nerve of groups of axons with different conduction velocities and different diameters followed the seminal discovery of a correspondence between axon caliber and sensory modality in the somatosensory system (Gasser and Erlanger, 1929; Heinbecker et al., 1933) and prompted the question of whether the different conduction velocity groups in the optic nerve also underpin parallel streams of visual information (see Stone, 1983). The presence of distinct groups of optic nerve fibers with different conduction velocities in response to electrical stimulation was later confirmed in the cat (Bishop and O’Leary, 1938). Subsequently, recording from within the dorsal lateral geniculate nucleus (dLGN), Bishop and McLeod (1954) showed that volleys arrived at different times (which they called *t*_1_ and *t*_2_) and each led to the generation of local field potential (LFPs), interpreted as corresponding postsynaptic responses of dLGN neurons (*r*_1_ and *r*_2_; Bishop and McLeod, 1954). The work of Stone and Hoffmann (1971) established a correspondence between dLGN neuron orthodromic latency and the antidromic latency to electrical stimulation of visual cortex (V1), thus demonstrating the segregation of pathways according to conduction velocity (i.e., axonal diameter) from retina to V1.

Parallel pathways can be distinguished by a number of visual response properties. In cat, linear of spatial summation of contrast over their receptive fields is the hallmark of X cells, while Y cells display non-linear spatial summation (Shapley and Hochstein, 1975; Shapley et al., 1981). In addition, X cells show sustained responses with high-spatial and low-temporal selectivity and low-contrast sensitivity, while Y cells group at the other end of the spectrum of these response properties (Cleland et al., 1971, 1973). X and Y cells can also be differentiated by the precision and reliability of their responses (Reinagel and Reid, 2000; Kumbhani et al., 2007) and the size of their receptive fields at matching eccentricities (Saul and Humphrey, 1990; Usrey and Reid, 2000; Xu et al., 2001; Weng et al., 2005).

Further parallel pathways exist in the magnocellular and parvocellular layers primate dLGN (Nassi and Callaway, 2009). Cells in the parvo and magnocellular layers of the LGN show unimodal distribution of visual response properties that are very similar to cat X and Y cells, respectively (Dreher et al., 1976; Sherman et al., 1976; Merigan and Maunsell, 1993; Levitt et al., 2001). Even though the strict application of linear spatial summation tests suggests the existence of two populations (linear and non-linear cells) in the magnocellular layers of primates (Shapley et al., 1981; Kaplan and Shapley, 1982), other studies have shown that the distribution of linearity is unimodal within magnocellular or parvocellular neurons (Levitt et al., 2001). In primates, processing is separated into at least one pathway for depth and motion and one for space and detail: the magnocellular and parvocellular pathways, respectively (Livingstone and Hubel, 1988; Callaway, 1998). Within the parvocellular population, color processing is also “parallelized” (Dacey, 2000; Nassi and Callaway, 2009).

Similar organization into parallel pathways has also been found in ferret, squirrel, and rat (reviewed in Van Hooser, 2007). However, little physiological evidence for parallel streams in mouse dLGN exists (Grubb and Thompson, 2003; Piscopo et al., 2013), despite evidence for multiple dLGN morphological populations (Krahe et al., 2011) and the clustering of visual response properties in cells of mouse primary V1 that suggests a parallel organization (Gao et al., 2010). Given the rising prominence of the mouse visual system as a tool for understanding visual processing (Niell and Stryker, 2008, 2010; Liu et al., 2010, 2011; Huberman and Niell, 2011; Niell, 2011; Polack et al., 2013), cortical structure and function (Sohya et al., 2007; Cardin et al., 2009; Sohal et al., 2009; Marshel et al., 2011; Adesnik et al., 2012; Bock et al., 2012; Olsen et al., 2012), and visually-guided behavior (Dombeck et al., 2007; Andermann et al., 2010; Busse et al., 2011; Lee et al., 2012; Carandini and Churchland, 2013; Saleem et al., 2013), it is important to characterize the output of mouse dLGN, if that output is organized into parallel streams when projecting to V1, and how that organization compares to what is known in other species. Towards this end, recent studies have identified direction and orientation selective cells (Marshel et al., 2012; Cruz-Martin et al., 2014) that may be analogous to koniocellular or W cell pathways and a diversity of response properties (Piscopo et al., 2013) in mouse dLGN.

Here, we present evidence for the existence of parallel streams in the retinogeniculate pathway of the mouse, however, the clustering of visual response properties of mouse dLGN neurons suggest a less segregated relay of visual information to primary V1. We recorded the spiking responses of single dLGN cells to electrical stimulation of the optic chiasm and to visual stimuli including spatiotemporal noise, drifting sinusoidal gratings, counterphased sinusoidal gratings, and a spatially-uniform flicker sequence. We first classified our cells based on the linearity of spatial summation to a counterphased modulating sinusoidal grating, and observed approximately 9:1 more X-like than Y-like cells, which we called linear and non-linear. However, these classes showed little difference in spatial and temporal and contrast response properties as well as in their receptive field parameters. Finally, with the possible exception of a subset of slower-responding linear cells, most cells recorded in mouse dLGN responded with approximately equal precision and reliability (Kumbhani et al., 2007).

## Materials and Methods

### Animal Preparation and Surgery

All procedures were done within the guidelines of the National Institutes of Health and were approved by the University of Pennsylvania Institutional Animal Care and Use Committee. Adult C57/B6 mice (8–24 weeks) were anesthetized with a high concentration of isoflurane (5%) and maintained with continuous inhaled isoflurane (0.8–1.2%). The depth of anesthesia was monitored using heart rate (maintained between 300 and 600 beats/min), pupil size, pinch reflex, and following the opening of the craniotomy, by the level of synchronous activity in the LFP. After placement in a stereotactic apparatus, eye moisture was maintained by application of a transparent lubricant and body temperature was maintained at 37°C by rectal monitoring and a heating pad (FHC Inc., Bowdoin, ME, USA). A 2-by-3 mm craniotomy was opened over dLGN. Following surgery, the entire stereotactic apparatus was rotated 60° to position the contralateral eye in front of the display screen.

### Electrophysiology

An array of four to six tetrodes (Thomas Recording GmbH, Giessen, Germany) arranged concentrically was inserted perpendicularly relative to the cortical surface. In both configurations, the tip-to-tip space between neighboring tetrodes was 254 μm. Individual tetrodes were 100 μm in diameter with a central contact at the tip 40 μm below three concentrically arranged contacts around the shaft 20 μm from each other. Signals were preamplified by the tetrode drive and amplified, individually filtered, and acquired at 30 kHz using a Cheetah 32 acquisition system (Neuralynx, Boseman, MT, USA). High-frequency spiking activity was isolated at each contact by filtering between 600 and 6000 Hz. A single channel from each tetrode was duplicated and filtered 0.1–375 Hz to record an LFP. Following a rest period of at least 30 min, each tetrode was lowered through the neocortex and hippocampus until audible modulation of background activity to a test stimulus was apparent. Tetrodes were further lowered until at least one isolatable unit appeared.

### Visual Stimuli

All visual stimuli were generated using the ViSaGe stimulus generation hardware (Cambridge Research Systems, Cambridge, UK) and a custom software package utilizing the accompanying MATLAB (Mathworks, Natick, MA, USA) toolbox. Stimuli were displayed on a 19-inch cathode ray tube monitor configured to refresh at 100 Hz at 600 × 800 resolution. This monitor was gamma-corrected using a luminometer and ViSaGe configuration software and placed 30 cm from the eye contralateral to the craniotomy. Full-screen stimuli covered approximately 70° of visual field. After tetrode insertion, the screen was set to a background of 50% luminance. Stimuli consisted of drifting sinusoidal gratings, stationary contrast-reversing gratings (i.e., counterphased), two-dimensional ternary white noise, and a spatially-uniform contrast modulating flicker stimulus. Counterphased sine-wave gratings were the size of the display (~70°), had variable spatial phase, and their contrast was reversed at 2 Hz following a square wave. Ternary white noise and spatially uniform flicker updated at 50 Hz. For ternary white noise the contrast each 50 × 50 pixel square was chosen for each frame independently of the previous frames and other pixels in that frame. Flicker stimuli consisted of a repeated sequence of contrasts; this sequence was generated by choosing randomly from a flat distribution of contrasts.

### Electrical Stimulation

Electrical stimuli were delivered through a bipolar stimulating electrode inserted proximal to the optic chiasm through independent burrhole craniotomies made with 500 μm of bregma. Each lead was connected to a stimulus isolation unit controlled by a Master-8 pulse stimulator (A.M.P.I., Jerusalem, Israel). Stimulation was monophasic and the duration was 50 μs. Initially large stimulus intensities (2 mA) were stepped down in ~0.1 mA increments in order to determine the sensitivity of components of the dLGN response.

### Spike Clustering and Data Analysis

Spike waveforms from each tetrode were clustered into individual units offline using a mixture of algorithmic and manual sorting (Spike- Sort3D, Neuralynx). Waveforms were initially sorted using KlustaKwik and subsequently manually refined. All clusters with spikes in the 0–1 ms bin of the interspike interval histogram were strictly rejected. To assess the quality of separation of the identified single units, we measured isolation distance and the L-ratio for each cluster, which indicate the distance of the center of the cluster from the noise and the quality of the moat around the cluster, respectively (Schmitzer-Torbert et al., 2005). Linearity of spatial summation was measured using the frequency components of the response to counterphased, stationary sinusoidal gratings (Shapley and Hochstein, 1975). The analyses of responses to drifting gratings and ternary white noise were performed as elsewhere (Denman and Contreras, 2014).

## Results

In order to investigate the organization of the mouse retinogeniculate pathway in relation to functional parallel streams, we recorded LFPs and single units from the dLGN using an array of independently-positionable tetrodes in isoflurane-anesthetized mice (*n* = 18). We studied responses to electrical stimulation of the optic chiasm and to a battery of visual stimuli. These recordings yielded 311 single units and 24 multi-unit clusters consisting of a mixture of spikes from several cells. Unless otherwise noted, the analyses described below were all performed on isolated single units.

### Electrical Stimulation of the Optic Chiasm

In the dLGN of cat (Bishop and McLeod, 1954) and primate (Reese and Cowey, 1990), the afferent fibers from retinal ganglion cells have a non-unimodal distribution of conduction velocities, which corresponds to the non-unimodal distribution of retinal axon diameters in the optic tract (Bishop, 1933, 1946; Bishop et al., 1953; Bishop and Clare, 1955; Guillery et al., 1982). Detailed anatomical characterization of C57/B6 retinal axons suggest a bimodal distribution of axonal diameters (Seecharan et al., 2003), but to our knowledge no study of mouse dLGN activity evoked by electrical stimulation of the optic nerve has been published.

**Figure 1:**
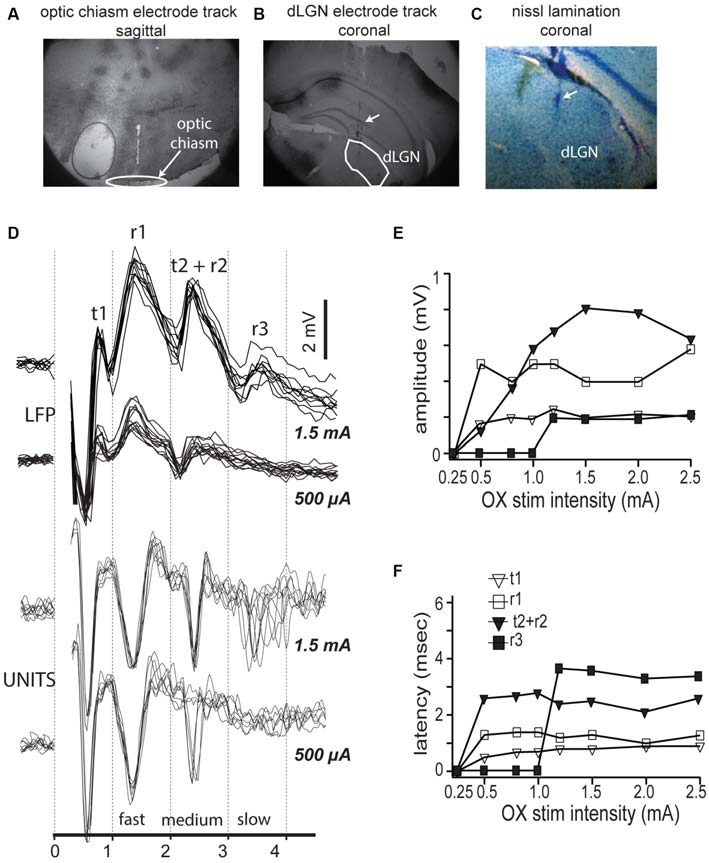
**Optic chiasm stimulation reveals three compound field responses and corresponding spike responses in mouse dorsal lateral geniculate nucleus (dLGN). (A)** Electrode track showing positioning of stimulating electrodes at optic chiasm fibers. **(B)** Electrode tracks showing multiple tetrode placement into dLGN. **(C)** Nissl stain of dLGN showing lack of obvious lamination. **(D)** Top traces, Local field activity in mouse dLGN following dLGN stimulation, at two stimulus intensities: 500 μA and 1.5 mA. Local field potential (LFP) shows four response components. Bottom traces, spikes recorded in LGN in response to same two intensities, overlaid traces to repeated stimuli show three latencies corresponding with the three LFP components. **(E,F)** Measurement of response component amplitudes **(E)** and latencies **(F)**.

To test for the presence of functionally distinct neuronal populations in mouse dLGN, we first analyzed the responses to electrical stimulation of the optic chiasm. We estimated the distance along the optic tract from the stimulating electrode to the dLGN to be 4.5 mm, according to the placement of the stimulating electrode just caudal to bregma (Figure 1A), the online 3D mouse brain atlas (Allen Institute 3D Connectivity Atlas) and published measurements of the mouse optic nerve (Kurimoto et al., 2010). We also verified the placement of our electrodes into dLGN histologically after each experiment (Figure 1B). We saw no obvious lamination in mouse V1 (Nissl stain, Figure 1C). Based on our distance estimation and the conduction velocities for the two primary axonal populations measured in other species (Gouras, 1969), 3.8 and 1.8 m/s, we expected to see electrically evoked responses with latencies of 1.2 and 2.5 ms. Bipolar LFP responses showed four overlapping peaks similar to those described in cat (Bishop and McLeod, 1954; their Figure 2A; here we follow their original nomenclature). An early positive-negative peak that corresponds with the arriving volley of fast conducting fibers, the *t*_1_ component, followed by the synchronous postsynaptic potential in dLGN neurons, the negative-positive potential *r*_1_. The subsequent large and less precise negative field (*r*_2_) corresponds with the postsynaptic potential triggered by the slower fibers, which in cats lead to a visible tract volley (*t*_2_; Figure 1D, bottom). Thus, both in mice and rats (Sefton and Swinburn, 1964), *r*_2_ and *t*_2_ appeared fused. Finally, at longer delays we see *r*_3_, which corresponds with postsynaptic potential of slower conducting fibers. The peak *r*_1_ reliably followed the presynaptic volley *t*_1_ at low stimulation intensities (Figure 1D, top), but *t*_2_, *r*_2_ and *r*_3_ were only present at higher stimulation intensities (Figure 1D, top), an intensity dependence relationship similar to that described by Bishop and McLeod (1954) and Bishop et al. (1959). This activation sequence was not modified by switching the stimulus polarity (not shown). Strength response curves for the different components revealed that the fastest component, *t*_1_ had the lowest stimulation intensity threshold (Figure 1E), and that *r*_1_ only occurred after *t*_1_, and *t*_2_ after *r*_2_, and *r*_3_ only occurred at higher intensities (Figure 1F).

**Figure 2:**
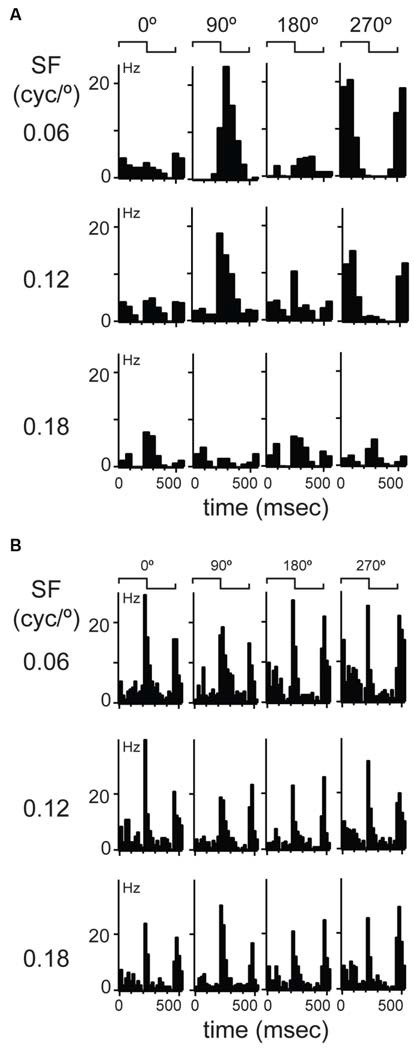
**Examples of linear and non-linear spatial summation in mouse dLGN. (A)** An example of a more common, linear summating unit. Cyclograms from peristimulus time histograms of responses to four spatial phases separated by 90° are shown in each row; rows show responses at different spatial frequencies indicated at left. Spike waveform on each wire shown at right along with a projection in cluster space showing cluster isolation. **(B)** An example of a unit showing non-linear spatial summation.

X and Y cells in cat (Cleland et al., 1971; Stone and Hoffmann, 1971), tree shrew (Sherman et al., 1975), as well as parvo and magnocellular cells in primate dLGN (Dreher et al., 1976; Sherman et al., 1976; Levitt et al., 2001), have statistically different mean response latencies to electrical stimulation of retinal axons. We examined the latency of dLGN spike responses to optic chiasm electrical stimulation (Figure 1D, bottom). We saw fast (~700 μs) spikes occurring at delays of 1.2, 2.2, and 3.5–4.0 ms after optic chiasm stimulation, consistent with spike latencies reported in cat (Hoffmann and Stone, 1971) and rat (Fukuda et al., 1979; Hale et al., 1979; Crunelli et al., 1987). Reversing the polarity of the bipolar stimulus changed the stimulus intensities necessary to elicit spike responses (not shown) but the responses had the same latencies and amplitudes.

### Classification of Units with the Modified Null Test

The observation of consistent response components in LFP and multiunit recordings in mouse dLGN suggests distinct populations of relay cells, in agreement with a previous anatomical study (Krahe et al., 2011). To classify dLGN units physiologically, we first utilized the modified null test (Enroth-Cugell and Robson, 1966; Cleland et al., 1971; Shapley and Hochstein, 1975). We presented stationary gratings with a sinusoidal modulation of contrast across space and a square modulation in time (counterphased, period = 0.5 s; contrast = 100%). The gratings were presented at 11 spatial phases, with 30° phase increments, and at four spatial frequencies (0.06 cycles/°, 0.12 cycles/°, 0.18 cycles/°, and 0.24 cycles/°). In cats, a dLGN cell is classified as an X-cell by the presence of at least one spatial phase that elicits no response to the temporal modulation of the grating. The presence of such null-phase indicates that the cell sums contrast inputs linearly over space. The majority of our cells had at least one null-phase at one of the tested spatial frequencies (277/311). We called these cells linear cells. We resisted the temptation of calling them X-like because, as will be shown below, their response properties were not clustered uniformly around expected values characteristic of X cells. The example dLGN neuron shown in Figure 2A showed the largest response at 0.06 cycles/° and at a 90° phase. This cell had two null-phases 90° away from the maximum, at 0 and 180°. Responses were robust at the 0.06 and 0.12 cycles/° but this cell did not respond at the highest spatial frequency of 0.18 cycles/°.

A subset of dLGN cells (34/311) did not display a null-phase, such as the example shown in Figure 2B, indicating that these cells do not perform linear summation of their inputs over space. Furthermore, at all spatial phases and all spatial frequencies, these cells responded to both contrast reversals during a stimulus cycle, thus leading to a response at twice the temporal frequency of the grating (Figure 2B). In cats (So and Shapley, 1979) and primates (Shapley et al., 1981; Kaplan and Shapley, 1982) these LGN cells are called Y cells. Here, we called them non-linear cells because, as will be shown below, their visual response properties were not uniformly consistent with this category in other species. The extracellular waveforms of putative linear and non-linear cells were not significantly different in amplitude of the rising phase, ratio of peak-to-trough, or the slope of the repolarization phase (data not shown).

### Linearity of Spatial Summation

**Figure 3:**
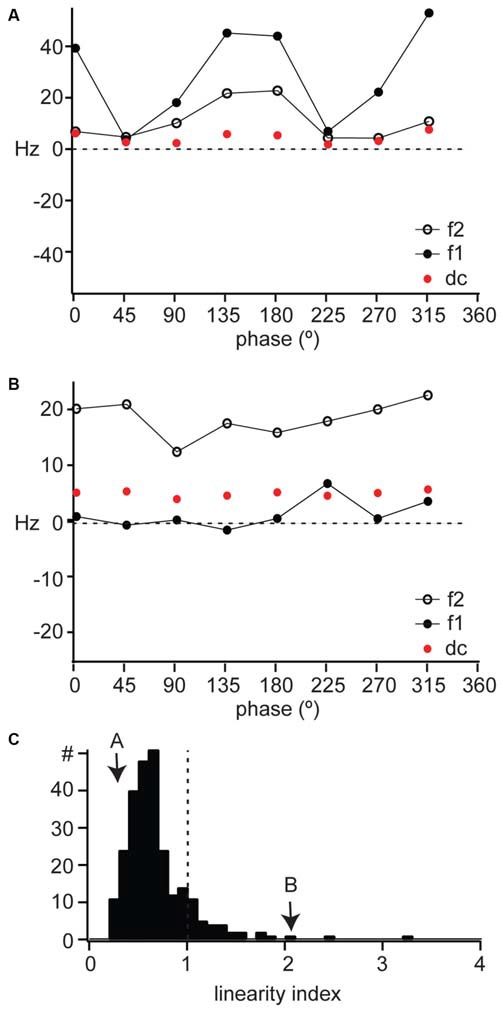
**Linearity of spatial summation in mouse dLGN. (A,B)** The DC, F1, and F2 components of the response to counterphased gratings across all spatial phases for the example cells shown in Figure 2. **(C)** The distribution of linearity index across our population of dLGN single units, with the examples in parts **(A)** and **(B)** indicated with arrows. A linearity index above 1 indicated non-linear spatial summation.

X cells respond to the contrast reversal of a sinusoidal grating at the modulation frequency of the grating (Shapley and Hochstein, 1975; So and Shapley, 1979; Kaplan and Shapley, 1982), so that their response is dominated by the fundamental frequency (F1, or first harmonic) of the stimulus. This modulation at F1 is greater than mean firing rate (F0 or DC). Furthermore, the F1 component of an X cell response is modulated sinusoidally as a function of the spatial phase of the stimulus. The example linear cell shown in Figure 2A had a sinusoidal modulation of its response F1 as a function of spatial phase (Figure 3A, filled circles), with a much smaller change in mean firing rate (Figure 3A, DC, red symbols). This unit’s response had a small F2 component (Figure 3A, open symbols) also modulated by the spatial phase, but overall the response was dominated by the F1 component.

Y cells respond to contrast reversal of the grating at twice its modulation frequency (Shapley and Hochstein, 1975; So and Shapley, 1979; Kaplan and Shapley, 1982). This leads to a response dominated by the second harmonic (the F2 component) of the modulation frequency of the grating. Furthermore, the F2 component is independent of spatial phase. For example, the non-linear unit illustrated in Figure 2B showed a response dominated by the F2 component (Figure 3B, open symbols), which was larger than both the F1 (Figure 3B, filled symbols) and DC (Figure 3B, red symbols) components and remained constant across spatial phases. The DC component of the response was also constant across spatial phases.

**Figure 4:**
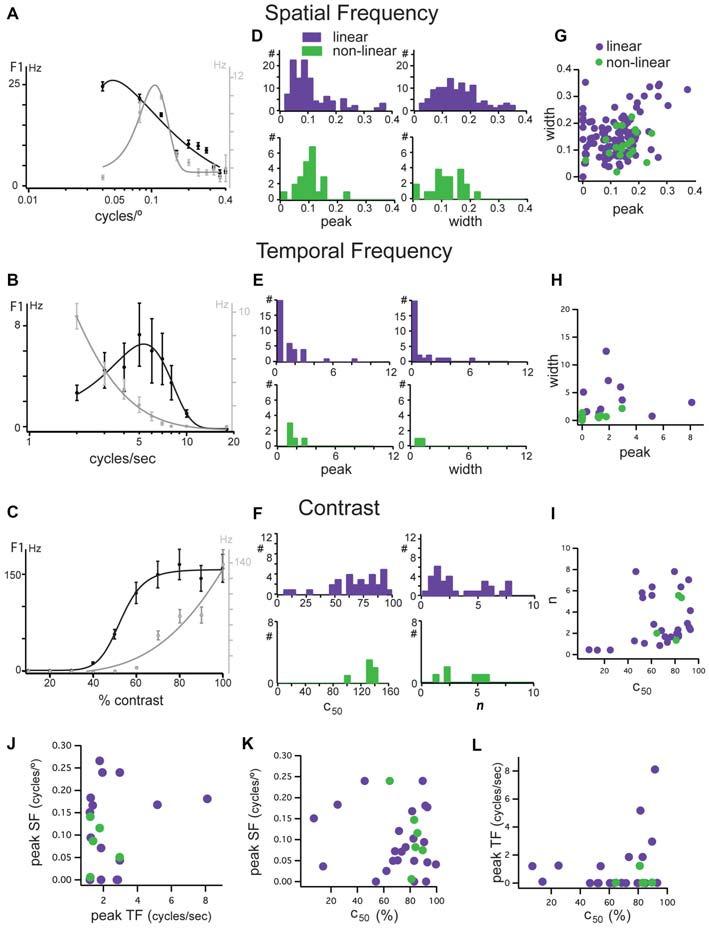
**Tuning characteristics of linear and non-linear units in mouse dLGN. (A–C)** Examples of opposing spatial frequency, temporal frequency, and contrast tuning. Two units shown, one a low-pass unit in black and bandpass in gray. Same two units in each panel. **(D)** Distributions of peak spatial frequency (left) and width of spatial frequency tuning (right) for cells classified as linear (purple bars) and non-linear (green bars) using the modified null test, measured from fits to spatial frequency tuning plots. **(E)** Distributions of peak temporal frequency (left) and width of temporal frequency tuning (right) for cells classified as linear (purple bars) and non-linear (green bars) using the modified null test, measured from fits to temporal frequency tuning plots. **(F)** Distributions of c_50_ (left) and *n* parameters (right) of contrast response functions for cells classified as linear (purple bars) and non-linear (green bars) using the modified null test. **(G)** Correlation of spatial frequency tuning width with peak spatial frequency, taken from fit parameters. **(H)** Correlation of temporal frequency tuning width with peak spatial frequency, taken from fit parameters. **(I)** Correlation of the slope and c_50_ of hyperbolic ratio fits of contrast response functions. **(J)** Correlation of peak spatial frequency with peak temporal frequency. **(K)** Correlation of peak spatial frequency with c_50_. **(L)** Correlation of peak temporal frequency with c_50_.

We measured the linearity of spatial summation for all units as the peak of the F2/F1 ratio across all spatial frequencies (Van Hooser et al., 2003). A ratio above 1 indicates non-linear spatial summation (Figure 3C, dotted line) and a ratio below 1 indicates linear summation. The linear unit in Figure 4A had a linearity index of 0.24 (Figure 3C, “A”) and the non-linear unit in Figure 4B had a linearity index of 2.0 (Figure 3C, “B”). Our population showed a unimodal distribution and was dominated by linear cells (277/311); we identify for the first time a population of non-linear cells in mouse dLGN (34/311).

### Response Properties of Linear and Non-linear Cells

In addition to the distinction based on response latency and linearity of spatial summation, functionally distinct dLGN populations in other species show differences in their contrast sensitivity and their selectivity to spatial and temporal frequency (Cleland et al., 1971; Sherman et al., 1975, 1976; Dreher et al., 1976; Derrington and Lennie, 1984; Price and Morgan, 1987; Livingstone and Hubel, 1988; Levitt et al., 2001). Typically, X cells respond better to higher spatial and lower temporal frequencies and have high contrast sensitivity, while Y cells prefer higher temporal and lower spatial frequencies and have high contrast sensitivity, though significant overlap between these pathways has also been reported (Bullier and Norton, 1979). We probed single units in mouse dLGN with a battery of gratings that varied in spatial frequency, temporal frequency, and contrast (Figure 4).

**Figure 5:**
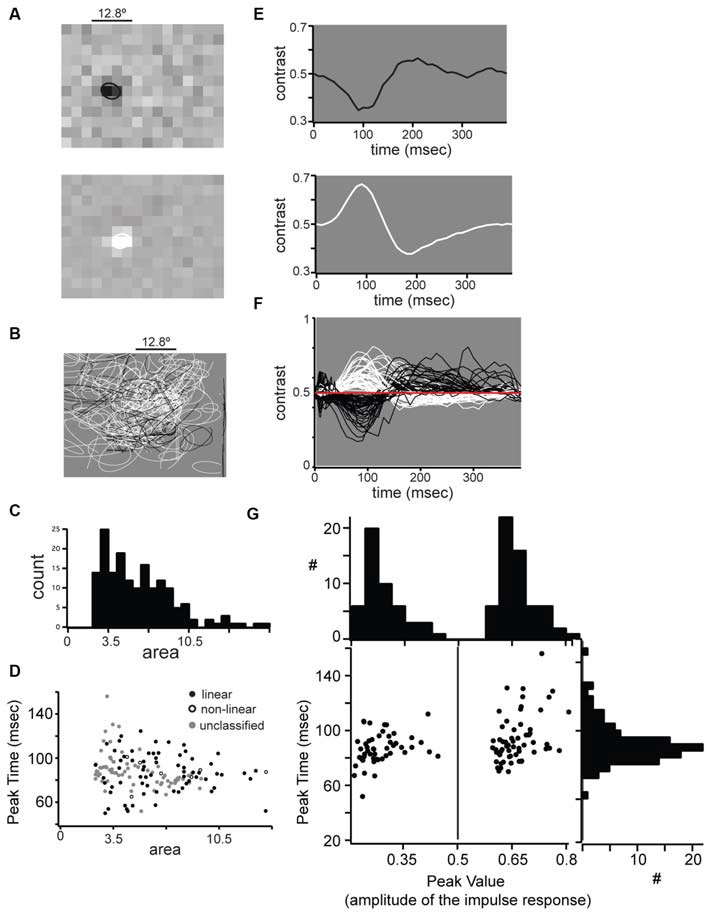
**Receptive field properties of single units in mouse dLGN. (A)** Example spatial receptive fields from an OFF-center (top) and OFF-center (bottom) cell. **(B)** All spatial receptive fields from our population. For each cell the 0.5 level contour from a two-dimensional Gaussian fit is shown. **(C)** Distribution of receptive field areas in mouse dLGN, calculated from two-dimensional Gaussian fit parameter. **(D)** No difference in linear, non-linear, and unclassified receptive field areas. **(E)** Impulse response function from the center of the example receptive fields shown in part **(A)**. **(F)** All impulse responses from our population. **(G)** Bimodal distribution of impulse response absolute maxima, but unimodal distribution of maximum time.

In some cells, we observed a clustering of response properties such as those exemplified by the two linear neurons in Figures 4A–C. The linear neuron depicted in black had a low-pass selectivity for spatial frequency with a peak response at 0.05 cycles/° (Figure 4A), a high temporal frequency preference with a peak at 5 cycles/s (Figure 5B) and high contrast sensitivity (c_50_ = 54%; Figure 4C). The linear neuron depicted in gray was band-pass for spatial frequency, with a higher peak spatial frequency of 0.12 cycles/° (Figure 4A), low-pass for temporal frequency with a peak response at 2 cycles/s (Figure 4B), and a higher contrast sensitivity (c_50_ = 89%; Figure 4C). While these cells seem to match X-like (the gray cell) and Y-like (the black cell) properties, both had null spatial phases to counterphased gratings and were classified as linear.

Analysis of the population revealed three important features: (1) In mouse dLGN, stimulus preferences were not correlated with null-test based classification. Distributions of peak spatial frequency (Figure 4D, left), spatial frequency bandwidth (Figure 4D, right), peak temporal frequency (Figure 4E, left), temporal frequency bandwidth (Figure 4E, right), the mid-saturation point (c_50_; Figure 4F, left), and the slope (*n*) of the contrast response function (Figure 4F, right) were not statistically different between linear and non-linear cells (*p* > 0.05, Wilcoxon rank test). Furthermore, unlike in other species, we found that both linear and non-linear cells span a broad range of stimulus preferences; (2) We did not observe a consistent correlation between the peak and the width of the spatial (Figure 4G) or temporal (Figure 4H) frequency selectivity, nor between the mid contrast and slope of the contrast response functions (Figure 4I), meaning that cells with high spatial and/or temporal frequency did not necessarily have narrower tuning curves; and (3) We did not observe a clustering of visual response properties, as shown by the lack of correlation between selectivity to spatial and temporal frequency or contrast sensitivity (Figures 4J–L). Due to the high degree of overlap in stimulus preferences between linear and non-linear cells it is likely that each of postulated parallel channels in mouse V1 (Gao et al., 2010) receive their thalamic inputs from a mixture of linear and non-linear dLGN cells.

### Receptive Field Properties

We mapped the RF of dLGN units with reverse correlation on spikes elicited by dense ternary noise (Figure 5). To quantify RF size we fit the RFs with a 2-dimensional Gaussian and used the square of σ as a measure of RF area; the example ON-center and OFF-center linear units in Figure 5A had RF center areas of 7.8 and 7.6°^2^, respectively. The areas of RF centers for the population span from 3.5 to 20.4°^2^ (Figure 5B) with a unimodal distribution and a median of 8.3°^2^ (Figure 5C). Thus, in mouse dLGN, linear and non-linear units could not be distinguished from each other, nor from unclassified units, based on RF size (Figure 5D).

The time course of the responses of ON- and OFF- cells was similar as shown by the impulse response of two example linear cells (Figure 5E) and the superimposed impulse responses of the population (Figure 5F). We compared the impulse response functions of ON- and OFF-center cells by plotting the peak amplitude vs. peak time (Figure 5G). While ON and OFF cells were distinguished by the polarity of the peak amplitude (along the *x*-axis), the distribution of peak times (along the *y*-axis) was unimodal with a mean of 83.9 ± 11.8 ms, showing that ON and OFF cells show similar response time course. Unlike previous reports (Piscopo et al., 2013), we observed transient and sustained temporal profiles from both ON and OFF cells. Furthermore, the time course of the impulse response did not distinguish between linear, non-linear or unclassified neurons (Figure 5G, distribution along the *y*-axis).

### Precision and Reliability

**Figure 6:**
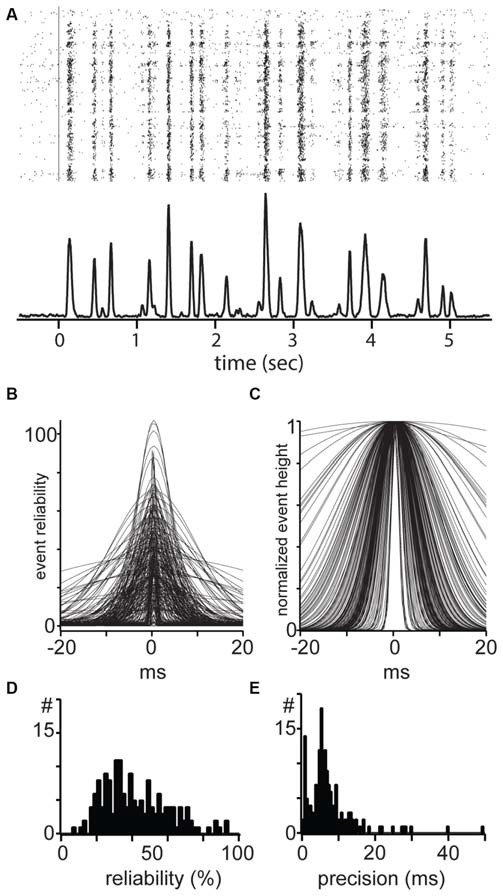
**Reliability and temporal precision of mouse dLGN single units. (A)** Example response of a mouse dLGN cell to spatially uniform flicker. **(B)** All events from all recorded mouse dLGN units to flicker stimulus; each identified event has been fit with a Gaussian, aligned, and overlaid. **(C)** All events, normalized to the maximum of each event to show the temporal precision of each event. **(D)** Distribution of event reliability. **(E)** Distribution of width from the Gaussian fit to each event.

In cats, Y cells have slightly higher precision and reliability than X-cells when tested with a stimulus with rapidly changing contrast (Reinagel and Reid, 2000; Kumbhani et al., 2007). Such differences are in part attributed to the higher temporal resolution of Y-cells in that species. We used a full screen flicker stimulus consisting of spatially homogeneous stimulus whose contrast varies rapidly (50 Hz), drawing from an even distribution of contrasts. Linear units responded robustly to repeated presentations of the same stimulus sequence (Figure 6A, raster plots), giving rise to clear distinguishable events in the accumulated PSTH (Figure 6A, bottom row, 1 ms bins). These events were at much higher firing rates than the background as seen by the period before time zero in the PSTH.

To estimate precision and reliability we identified events from the PSTH based on a threshold and fit each event with a Gaussian. These fits for all the events and from all cells are shown in Figure 6. Here, event reliability is measured as the height of the fit, which represents the percent of trials in which a spike occurred (Figure 6B). After normalizing for differences in reliability (Figure 6C), we used the width of the events as an estimate of spike precision of the total spikes in the event.

For each cell, we took the percent of stimulus presentations in which there was at least one spike in the event window (±30 ms around the peak). This measure of reliability quantifies the reproducibility of the entire spike train in the response, independent of variability within each event. The population of dLGN cells was distributed along a range of reliability from 3% to 94%, with a median of 31.6% and mean of 35.5 ± 23.0% (Figure 6D). The distribution of precision of all events in all cells spanned the range of 0.5–50 ms and showed a unimodal distribution with a median of 7.9 ms and a mean of 8.8 ± 6.6 ms (Figure 6E). In conclusion, we did not observe a difference in precision or reliability between linear and non-linear cells.

## Discussion

In this study, we have used electrical and visual stimulation to study parallel processing in mouse dLGN and find evidence for parallel pathways in only partial homology with cats and monkeys. We find that in the mouse, three distinct populations of dLGN neurons could be distinguished on the basis of the latency of response to electrical stimulation of the optic chiasm. We find that mouse dLGN is dominated by neurons that perform linear spatial summation, though we do observe a subpopulation with non-linear spatial summation properties and frequency doubling. Unlike in cats and primates, linearity of spatial summation did not correlate with RF size, response precision, spatial and temporal selectivity, or contrast sensitivity.

### Retinal Basis of Mouse dLGN Parallel Streams

In both primates and cats, parallel processing streams are established in the retina. In the macaque, retinal ganglion cells can be distinguished morphologically; the predominant class is the “midget” (or type III or B-type) ganglion cell, which is smaller than the “parasol” (or type II or A-type) ganglion cell (Levanthal et al., 1981; Watanabe and Rodieck, 1989). These cells are also distinguished by their response properties: midget cells tend to have sustained responses to flashed spots, whereas parasol cells display transient responses (De Monasterio and Gouras, 1975). In cats, X and Y type retinal ganglion cells are distinguished by morphology, and by the linearity of spatial summation (Enroth-Cugell and Robson, 1966) and the transient or sustained nature of their responses (Cleland et al., 1971). Like primate and cat retinae, mouse retina contains >20 retinal ganglion cell types (Sun et al., 2002; Völgyi et al., 2009). Morphometric analyses of soma size and dendritic field shape suggest that these types include homologs of A and B type primate retinal ganglion cells and X and Y type cat cell ganglion cells. Recordings from mouse retina validate this morphological evidence: sustained and transient ganglion cells have been observed in the mouse retina (Balkema and Pinto, 1982). In addition, non-linear spatial summation is observed in a subset of mouse retinal ganglion cells (Stone and Pinto, 1993). It is therefore reasonable to hypothesize the continuation of parallel streams into mouse dLGN. Indeed, the percentage of X-like ganglion cells reported by Stone and Pinto (1993); 87% agrees well with percentage of linearly summating cells in mouse dLGN (89%; present study).

### Similarities between Mouse dLGN and those of other Mammals

We noticed several similarities between dLGN of mouse and dLGN of domestic cat. Multiple component field responses were first observed following optic chiasm stimulation in the cat (Bishop and McLeod, 1954; Bishop et al., 1959). Similarly, we observed compound responses in the field potential in mouse dLGN. While we were unable to measure spike latencies from isolated single units, we did see multiple compound high-frequency spikes with distinct latencies. Direct comparison of the observed mouse potentials with those from other species (Bishop and McLeod, 1954; Hale et al., 1979) is complicated by the small size of structures in mouse and the arrangement of cat and primate dLGN in distinct horizontal layers with vertical optic tract input. Stimulation of mouse optic chiasm may spread to the nearby optic nerve or optic tract and the volleys may arrive from both sides with slightly different delays. In the mouse, the lack of lamination may result in different arrangement of inputs and differences in waveforms compared to those of cat and monkey.

Furthermore, in cat retina (Enroth-Cugell and Robson, 1966) and dLGN (Shapley and Hochstein, 1975) cells have been classified on the basis of linearity vs. non-linearity of spatial summation within their receptive fields. We were able to identify 11% of mouse dLGN units as non-linear. The encounter rate of non-linear cells in the mouse dLGN (and retina) is thus much lower than encounter rate of Y cells (defined on other grounds than non-linearity of spatial summation) in dLGN of cats (48%; Sireteanu and Hoffmann, 1979), substantially lower than encounter rate of Y cells (defined on the basis of non-linearity of spatial summation) in dLGN of ferrets (23%; Price and Morgan, 1987) as well as substantially lower than encounter rate of Y-like cells (defined on other grounds than non-linearity of spatial summation) in dLGN of rats (27%; Hale et al., 1979).

Both of these factors, conduction velocity and linearity of spatial summation, can also distinguish streams in the macaque retinogeniculate pathway. In macaque a 25% subset of magnocellular cells are non-linear (Kaplan and Shapley, 1982), making the total percentage of non-linear cells ~8%, a number closer to the observed frequency in mouse. Like the percentage of linear cells we observe in mouse dLGN, and the number of B-type ganglion cells in mouse, the percentage of midget cells in the macaque retina is ~90% (Dacey, 1994). The great majority of macaque magnocellular and virtually all macaque parvocellular cells are reported to exhibit linear spatial summation (e.g., Kaplan and Shapley, 1982). Nevertheless, macaque’s magnocellular dLGN neurons could be easily distinguished from the parvocellular neurons on the basis of their spatial frequency preferences and contrast sensitivity (Livingstone and Hubel, 1988), By contrast, we did not see obvious separation of spatiotemporal profiles within the mouse dLGN linear population. However, it should be noted that the clear-cut separation in spatial frequency preferences between the magnocellular and parvocellular cells in macaque’s dLGN applies only when comparisons are made between magnocellular and parvocellular cells with receptive field at the same eccentricities. Mice, unlike virtually all primates, do not have fovea and there is very shallow center-periphery gradient in the density of their retinal ganglion cells density (Dräger and Olsen, 1981). Furthermore, in mouse, unlike in primates, neither somal sizes nor dendritic tree sizes of retinal ganglion cells increase substantially with lowering ganglion cell density (Sun et al., 2002). Thus, it is very unlikely that in the mouse, eccentricity differences in the retinal ganglion cell density would create substantial differences in spatio-temporal profiles of dLGN cells with receptive fields in different parts of the visual field.

Whether the ~90% of linearly responding cells in mouse dLGN more closely resemble magno or parvocellular cells is not immediately clear. Unlike macaque magnocellular and parvocellular populations, which are linear in spatial summation (Kaplan and Shapley, 1982) but identifiable on the basis of spatial frequency preference and contrast sensitivity (Livingstone and Hubel, 1988), we saw no obvious separation of spatiotemporal profiles within the mouse dLGN linear population. However, it should be noted that this separation in macaque depends strongly on eccentricity, which we did not account for here. Although mice do not have a fovea, there are differences in retinal ganglion density that may create differences in tuning across visual space (Bleckert et al., 2014) and very recently, Zhang et al. (2015) reported that the spatial frequency cut-offs of mouse V1 neurons with receptive fields in the upper visual field (where mouse aerial predators are likely to lurk) is substantially higher than that of V1 neurons with receptive fields in the lower visual field. It should further be noted that overlap in the spatial and temporal tuning properties has also been reported in the visual pathways of squirrels (Van Hooser et al., 2003), rats (Hale et al., 1979), rabbits (Swadlow and Weyand, 1985), cats (Bullier and Norton, 1979), galagos (Irvin et al., 1993) and even macaques (Hicks et al., 1983). We did not explicitly measure transience, but the qualitative transience of responses in our population (e.g., Figure 2) leads us to hypothesize that mouse dLGN is dominated by magnocellular-like cells.

Our results in mouse dLGN are similar to those seen in rat dLGN (Hale et al., 1979), where three groups of cells can be distinguished by conduction velocities of their retinal afferents but not the sizes of their receptive fields. It has been argued that this homogeneity indicates a lack of an X-like pathway and dominance of Y-like and W-like pathways. Further, some anatomical evidence points to the lack of an X-like pathway in rats (Reese, 1988). It is possible that our population of linear cells is an entirely W-like population, though based on the receptive field shape and response dynamics to gratings we believe it more likely our linear cells are comprised of a mix of X-like and W-like cells.

### W-like and Koniocellular-like Responses in Mouse dLGN

In both cat and macaque a third, somewhat catch-all, class of geniculate cells contains a diversity of response properties including orientation selective responses (LeVay and Ferster, 1977; Hendry and Reid, 2000). Here, we see some evidence for orientation biased responses in mouse dLGN, but more complete and convincing descriptions of orientation selective responses in mouse dLGN have been published elsewhere (Huberman et al., 2009; Krahe et al., 2011; Piscopo et al., 2013; Scholl et al., 2013; Cruz-Martin et al., 2014). The shell region of mouse dLGN appears to be somewhat analogous to koniocellular and W cell pathways in other species; most notably, it relays direction selectivity circuit from the retina to superficial layers V1 (Cruz-Martin et al., 2014). The mouse does seem to contain a third pathway, how much of these mixed koniocellular and W streams are apparent in mouse dLGN is far from resolved by this or any other mouse studies.

### Major Differences between the Mouse dLGN and those of Cats and Macaques

While the organization of mouse dLGN contains several homologies to the macaque system, it must also be noted that there are also several major differences. The most obvious difference is the gross laminar organization: while in macaque’s dLGN cells cluster in several distinct layers with smaller numbers distributed in the interlaminar zones, mouse dLGN, like dlGN of rat (Reese, 1988) is not layered (Paxinos and Franklin, 2004). There is some organization in mouse dLGN, with W-like dendritic morphologies in a dorsal shell and X-like morphologies in a core (Krahe et al., 2011), but this pales in comparison to the organization of both cat and macaque dLGN.

We saw little difference in receptive field size between linear and non-linear cells in mouse dLGN, whereas Y and magnocellular cells tend toward larger receptive fields than X and parvocellular cells, respectively (Saul and Humphrey, 1990; Usrey and Reid, 2000; Xu et al., 2001; Weng et al., 2005). Several factors may contribute to our inability to see differences in receptive field size. Here, we have combined cells from across retinotopic positions; as receptive field size is correlated with eccentricity, different eccentricities with both the linear and non-linear samples could be a factor. In addition, the large spatial scale of the mouse system could limit our ability to fully stimulate very large receptive fields because of the limits of our stimulus monitor. To resolve this, measurement of eye position for display position and very large displays corrected for distortions may be required. Finally, color based parallel pathways within the parvocellular system are well described (Dacey, 2000), and while the mouse expresses two cone opsins and possesses color-based circuitry in the retina (Brueninger et al., 2011; Baden et al., 2013), little is known about parallel color pathways mouse dLGN. Further work investigating transience and opsin-specfic responses should help identify a parvocellular-like pathway in mouse dLGN, if one exists.

## Author Contributions

DJD designed experiments, performed research and data analysis, and wrote the manuscript. DC designed experiments and wrote the manuscript.

## Funding

This work was supported by the National Eye Institute-National Institutes of Health (Grant R01 EY020765 and Vision Training Grant 2T32EY00735).

## Conflict of Interest Statement

The authors declare that the research was conducted in the absence of any commercial or financial relationships that could be construed as a potential conflict of interest.
